# Clinical significance of cancer specific methylation of the *CDO1* gene in small bowel cancer

**DOI:** 10.1371/journal.pone.0211108

**Published:** 2019-01-24

**Authors:** Keita Kojima, Takatoshi Nakamura, Yosuke Ooizumi, Kazuharu Igarashi, Toshimichi Tanaka, Keigo Yokoi, Satoru Ishii, Nobuyuki Nishizawa, Hiroshi Katoh, Yoshimasa Kosaka, Takeo Sato, Masahiko Watanabe, Keishi Yamashita

**Affiliations:** 1 Department of Surgery, Kitasato University School of Medicine, Sagamihara, Kanagawa, Japan; 2 Division of Advanced Surgical Oncology, Department of Research and Development Center for New Medical Frontiers, Sagamihara, Kanagawa, Japan; University of South Alabama Mitchell Cancer Institute, UNITED STATES

## Abstract

Although small bowel cancer (SBC) is extremely rare, its prognosis is poor, and molecular mechanism of the SBC development remains unclear. The aim of our study is to elucidate whether DNA methylation of the promoter region of the cancer-specific methylation gene, *cysteine dioxygenase 1* (*CDO1*), contributes to the carcinogenic process in SBC. The study group comprised patients with 53 patients with SBC, 107 colorectal cancer (CRC), and other rare tumors of the small intestine such as 4 malignant lymphomas, 2 leiomyosarcomas, and 9 gastrointestinal stromal tumors. We analyzed the extent of methylation in each tissue using quantitative TaqMan methylation-specific PCR for *CDO1*. Significantly higher *CDO1* methylation was observed in cancer tissues compared with non-cancerous mucosa of the small intestine (ROC = 0.96). Among the various clinicopathological factors, positive correlation of *CDO1* methylation with tumor diameter was observed (R = 0.31, p = 0.03), and the *CDO1* methylation level was a possible prognostic factor for relapse-free survival (p = 0.09). Compared with CRC, SBC had a significantly poorer prognosis (p = 0.007) and displayed a significantly higher *CDO1* methylation level (p < 0.0001). Intriguingly, especially in pStage I/II, there were robust prognostic difference between SBC and CRC (p = 0.08 / p < 0.0001), which may reflect *CDO1* methylation status (p = 0.02 / p = 0.001). Among small bowel tumors, *CDO1* methylation in SBC was higher in order of malignant lymphoma, cancer, and leiomyosarcoma/GIST (p = 0.002) by ANOVA. The *CDO1* gene shows extremely cancer-specific hypermethylation, and it can be a prognostic marker in SBC.

## Introduction

The small intestine consists of the duodenum, jejunum, and ileum, and the total mucosal surface area accounts for about 90% of the total gastrointestinal tract [1]. Nevertheless, small bowel cancer (SBC) is markedly less frequent than other gastrointestinal cancers and it is a very rare disease [1–3]. Its global incidence has been reported as fewer than 1.0 per 100,000 people, when age-standardized to the world population [1, 4]. The general interpretation as to why the incidence of SBC is low is as follows: there is an absence of genetic damage accumulation due to the rapid metabolism of the small intestinal epithelium; there is mucosal immune surveillance by small intestinal lymphatic tissue; and the small bowel avoids contact with carcinogens in food by not stagnating intestinal contents [5, 6]. In the clinical setting, the use of the small intestine endoscope has become prevalent for examination of the small bowel, and in recent years there is a concern regarding an increase in the number of SBC patients [7]. In addition, according to the statistics of small intestinal tumors in the United States in 2009, the 5-year survival rate of patients with SBC who underwent surgical treatment is as low as 32.5%, and has barely changed in the 20 years from 1985 to 2004 [8]. Normal colon and small intestine accumulate similar numbers of somatic mutations with aging [9]. However, due to its very low frequency of occurrence, studies to understand molecular carcinogenesis are still not satisfactory in SBC.

DNA methylation is a promising disease marker. DNA methylation is a stable modification, and since it is very stable compared to RNA and protein, it is useful for analysis of tissue specimens. For these reasons, DNA methylation is a popular marker in research on cancer [10]. As a methylation abnormality in SBC, it has been found that the CpG island methylator phenotype occurs to 27%. This is almost the same as colorectal cancer [11, 12]. It has been reported that *MINT1*, *MINT2*, *MGMT*, *MLH1*, *p16*, *HPP1*, *APC*, etc. show methylation abnormalities in SBC [13, 14].

*CDO1* was identified as a methylation-specific gene in human cancer by a methylation gene identification method that used a pharmacological unmasking microarray method [15, 16]. *CDO1* functions as a tumor suppressor gene in various cancer cells. Methylation of the promoter region of the *CDO1* has been reported in a number of cancers including breast cancer, esophageal cancer, gastric cancer, and colon cancer [15, 17–21], however there has no report with regard to SBC.

CDO1 is a nonheme iron enzyme, which converts cysteine to cysteine sulfinic acid (CSA) [15, 22, 23]. CSA inhibits the outflow of protons from the mitochondria to intracellular compartments and maintains the mitochondrial membrane potential [24]. On the other hand, the production of glutathione from cysteine is suppressed, leading to an increase in reactive oxygen species and promoting apoptosis [25].

In this study, we for the first time investigated methylation abnormality of the *CDO1* and its association with the clinicopathological features of SBC, and the possibility that it might function as a SBC biomarker.

## Materials and methods

### Patients

Fifty-three patients who underwent surgery for the diagnosis of small bowel adenocarcinoma at Kitasato University Hospital from January 1, 1988 to July 31, 2016 were selected. Fifty-three cancer tissues and 34 non-cancerous mucosa that were available in the same patient were analyzed. In order to analyze whether it is a cancer-specific phenomenon also in the small intestine and to clarify the relationship between *CDO1* promoter methylation and protein expression, we used non-cancerous mucosa collected from a part close to cancerous tissue. Twenty cases of small bowel adenomas were also used.

Two patients were treated with neoadjuvant chemotherapy and 29 patients were treated with adjuvant chemotherapy. In 13 cases (24.5%), metachronous or a simultaneous double cancer (gastric cancer, colon cancer, renal cancer, ovarian cancer, uterine cancer, thyroid cancer, malignant thymoma) was observed. Other clinicopathological details are shown in S1 Table.

For comparison with SBC tissue, colorectal cancer, small intestinal malignant lymphoma, leiomyosarcoma, and gastrointestinal stromal tumors (GIST) were also selected. Colorectal cancer tissue was obtained from 107 patients who underwent surgery and did not receive neoadjuvant chemotherapy from January 1 to December 31, 2000. The details of the patients with colorectal cancer are as follows: 27 patients were pStage I; 31 were pStage II; 30 were pStage III; and 19 were pStage IV. Other small intestinal tumor tissue was obtained from patients who underwent surgery without neoadjuvant chemotherapy from 1988 to 2014. Four patients had small intestinal malignant lymphoma, 2 had small intestinal leiomyosarcoma and 9 had small intestinal GIST. All specimens used were formalin-fixed and paraffin-embedded (FFPE) tissues.

This research was a retrospective study and comprehensive consent was obtained in writing from all patients for sample collection. This study was conducted in accordance with the Declaration of Helsinki and was based on the approval of the Kitasato University Medical Ethics Organization. The approval number is B17-007.

### DNA purification from tissue and bisulfite treatment of DNA

Tissue sections were hematoxylin-eosin stained and the target tissue was excised with a scalpel. Thereafter, the genomic DNA was extracted using the QIAamp DNA FFPE Kit (Qiagen Sciences, Hilden, Germany). Bisulfite treatment was carried out using the EZ DNA Methylation-GoldTM Kit (Zymo Research, Orange, CA).

### Quantitative-methylation-specific PCR (Q-MSP)

Quantitative TaqMan methylation-specific PCR (Q-MSP) was conducted using iQ Supermix (Bio-Rad) in triplicate on the iCycler iQTM Real-Time PCR Detection system (Bio-Rad). Q-MSP was done at 95°C for 3 min, followed by 40 cycles of 95°C for 20 s, annealing temperature (60°C) for 30 s, and 72°C for 30 s in a 25-μL reaction volume containing 1 μL bisulfite-treated genomic DNA, 300 nmol/L of each primer, 200 nmol/L fluorescent probe, and 12.5 μL iQTM Supermix. The methylation-positive control used DLD1 cells, and the negative control used HepG2 cells. PCR conditions and the sequences of the primers and probes are provided in S2 Table. The methylation value (TaqMeth V) was defined as the quantity of fluorescence intensity derived from promoter amplification of the positive control gene divided by the fluorescence intensity from β-actin, which was then multiplied by 100.

### Cell lines

The colorectal cancer cell line DLD1 was kindly provided by the Cell Resource Center of the Biomedical Research Institute of Development, Aging and Cancer, Tohoku University (Sendai, Japan). The hepatocellular carcinoma cell line HepG2 was purchased from the RIKEN BioResource Centre (Ibaraki, Japan). DLD1 cells were grown in RPMI 1640 medium (GIBCO, Carlsbad, CA), and HepG2 cells were grown in DMEM (GIBCO). All media contained 10% fetal bovine serum and penicillin-streptomycin (GIBCO).

### Clinicopathological factors

The clinicopathological factors evaluated were as follows: age, sex, tissue type (differentiated types: tubular adenocarcinoma and papillary adenocarcinoma; undifferentiated types: poorly differentiated adenocarcinoma, mucinous adenocarcinoma and signet ring cell carcinoma), tumor location, tumor diameter, pathological depth of tumor invasion (pT), pathological lymph-node metastasis (pN), clinical distant metastasis (cM), pathological staging classification (pStage), lymphatic invasion (ly), and vascular invasion (v) according to the seventh edition of the American Joint Committee on Cancer/International Union Against Cancer staging system.

### Immunostaining of CDO1

FFPE tissue blocks were cut into thin sections (4 μm thick) that were then deparaffinized with xylene and dehydrated through a stepwise series of ethanol. For antigen activation, samples were immersed in pH 6 citrate buffer and boiled in a microwave for 15 minutes. For inactivation of endogenous peroxidase, it was immersed in 3% aqueous hydrogen peroxide for 30 minutes. The primary antibody reaction was performed using the rabbit CDO1 polyclonal antibody (12589-1-AP) (proteintech, Rosemont, USA; 1:100 dilution) and incubated overnight at 4°C. The secondary antibody reaction was performed using the Histofine Simple Stain MAX-PO(MULTI) kit (Nichirei, Tokyo, Japan) according to the manufacturer’s protocol. ImmPACT DAB (Vector Laboratories, Inc, Burlingame, CA) was used for 5 minutes to develop color. Mayer's Hematoxylin Solution was used for nuclear staining.

### Statistical analysis

The relationship between the TaqMeth V and clinicopathological factors was analyzed using Student's t-test, the chi-squared test, and Tukey's honest significant difference test and variance, as appropriate. The Kaplan-Meier method was used to estimate cumulative 5-year overall survival (OS) and relapse-free survival (RFS), and statistical differences were tested by the log-rank test. OS and RFS were measured from the date of surgery to the date of events or the last follow-up. Variables suggested to be prognostic factors on univariate analysis were subjected to multivariate analysis using a Cox proportional hazards regression model. P < 0.05 was considered statistically significant. P = 0.05 to 0.10 was defined as marginally significant. All statistical analyses were performed with SAS software package JMP, version 11 (SAS Institute Inc., Cary, NC, USA).

## Results

### Cancer specific methylation of the *CDO1* gene promoter region in SBC tissue

**Fig 1:**
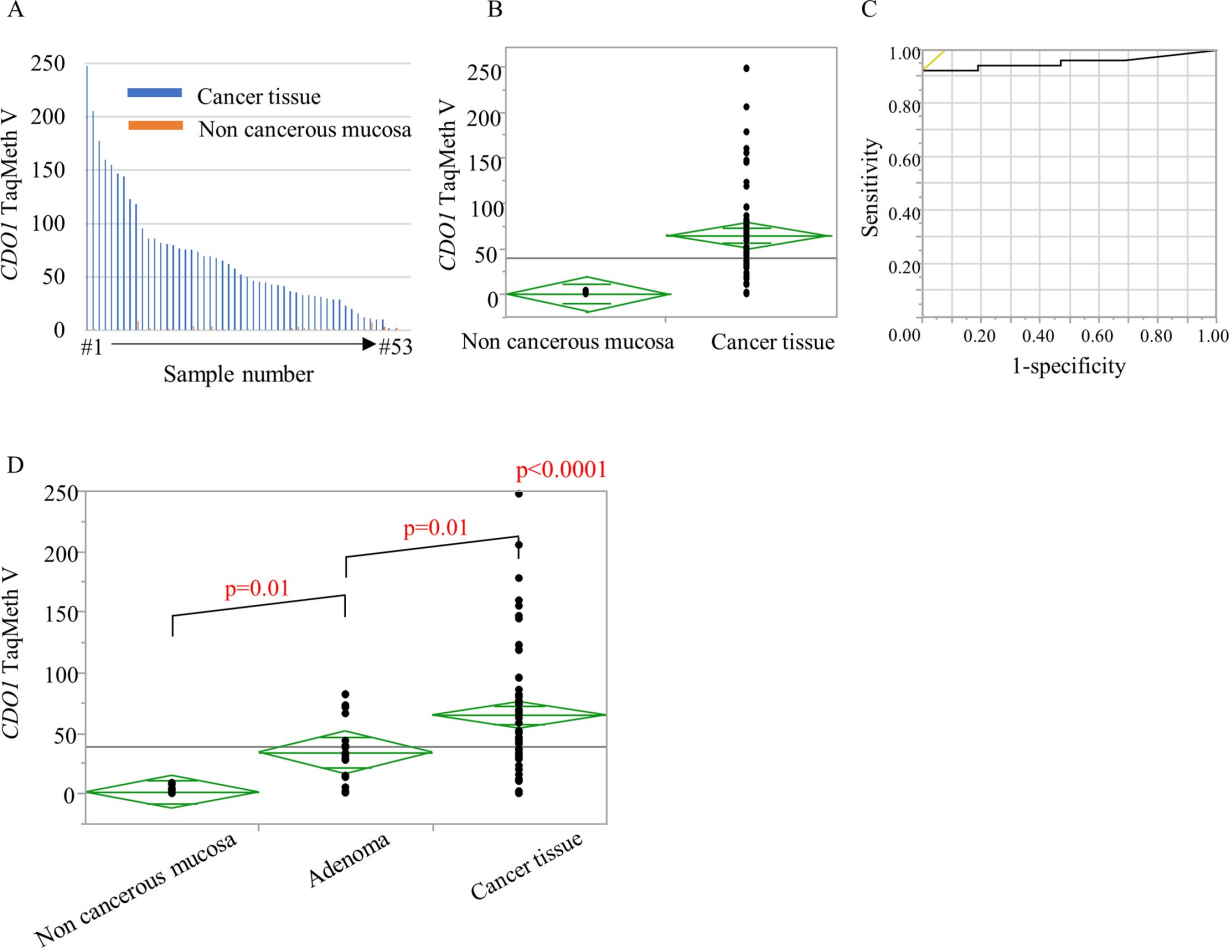
Methylation abnormality of the *CDO1* gene promoter region in small bowel cancer tissue. **A**, **B**: Comparison of the TaqMeth V of small bowel cancer tissue and small intestine non-cancerous mucosa. Significantly higher methylation was found in cancer tissues. Fig A shows TaqMeth V of cancerous tissues and its corresponding non-cancerous mucosa. **C**: ROC curve to distinguish between cancer tissue and non-cancerous mucosa. The cutoff value of TaqMeth V was 4.09 (AUC = 0.96; Sensitivity, 100%; Specificity, 92.5%). D: Comparison of TaqMath V between non-cancerous mucosa, small bowel adenoma and small bowel cancer tissue. TaqMath V showed a stepwise rise with increasing atypia.

Methylation of the *CDO1* gene in SBC tissue and corresponding non-cancerous mucosa was analyzed. The TaqMeth V in SBC tissue was 64.9 ± 54.2 and that in small intestine non-cancerous mucosa was 0.9 ± 1.2. SBC tissue showed significantly higher methylation than that of the non-cancerous mucosa (p < 0.0001) (Fig 1A and 1B).

We next determined if a cutoff value that could distinguish between the two types of tissue could be obtained by using a receiver operating characteristic (ROC) curve. The cutoff value of TaqMeth V (area under the curve (AUC) = 0.96; Sensitivity 92.5%; Specificity 100%) was 4.09 (Fig 1C).

Next, we evaluated for small bowel adenomas. Due to rare cases of small bowel adenomas, 20 cases were analyzed in total (11 duodenal adenomas, 4 jejunal adenomas, 5 ileal adenomas). The TaqMeth V in small bowel adenoma was 33.9 ± 24.1. In comparison with TaqMath V, small bowel adenoma showed significantly higher values than non-cancerous mucosa (p = 0.01). Similarly, TaqMath V of small bowel adenoma was lower than SBC tissue (p = 0.01) (Fig 1D). The cutoff value of TaqMeth V that could distinguish between non-cancerous mucosa and small bowel adenoma was 4.97 (AUC = 0.96; Sensitivity 90.0%; Specificity 94.1%). Similarly, the cutoff value of TaqMeth V that could distinguish between small bowel adenoma and SBC tissue was 41.1 (AUC = 0.68; Sensitivity 62.3%; Specificity 75.0%).

### Correlation of *CDO1* methylation with clinicopathological characteristics in SBC

**Fig 2:**
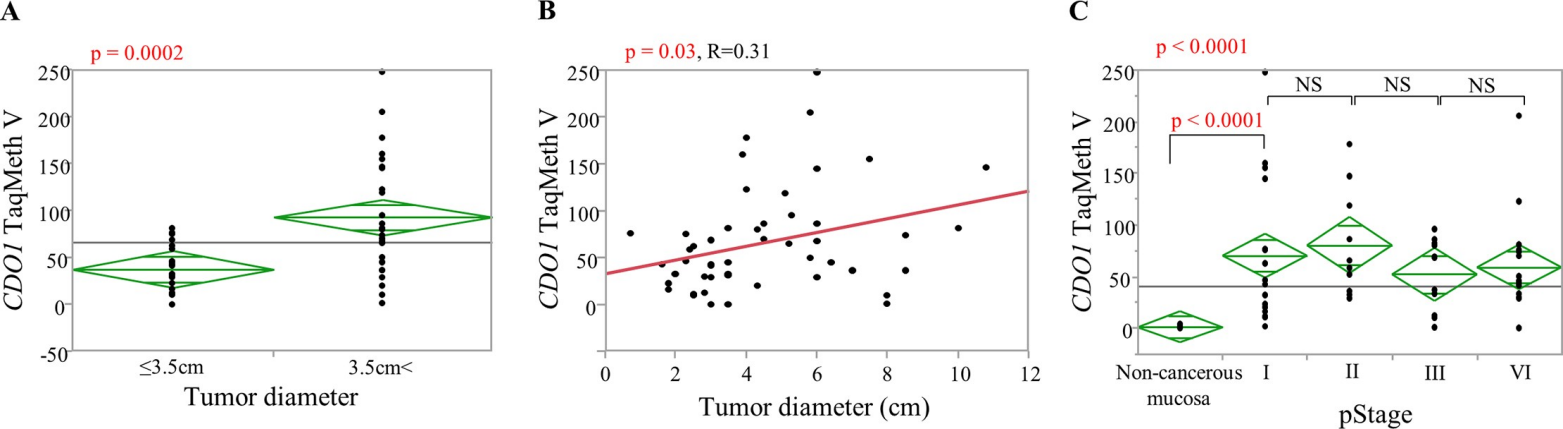
Relationship of methylation abnormality of the *CDO1* gene in small bowel cancer tissue with clinicopathological features. **A**: Result of a t-test on tumor diameter (cutoff value = 3.5 cm). Significantly high methylation was observed in the group with a tumor diameter that was larger than 3.5 cm. **B**: Relationship between tumor diameter and the *CDO1* TaqMeth V. As the tumor diameter increased, methylation was high. **C**: Comparison of the TaqMeth V in each stage. There was a significant difference between non-cancerous mucosa and pStage I. But, there was no significant difference in the TaqMeth V between stages. High methylation was found from Stage I.

Analysis of the relationship between *CDO1* methylation abnormality of SBC tissue and clinicopathological factors revealed a positive correlation of the TaqMeth V with tumor diameter (R = 0.31) and a significant difference in *CDO1* methylation was observed between groups with high and low tumor diameter when the cutoff value of the tumor diameter was 3.5 cm (p = 0.0002). There was no significant difference in the degree of *CDO1* methylation according to Stage, and *CDO1* was highly methylated from the early stages. As a result, there was significant difference between early stage (pStage I) and non-cancerous mucosa. In addition, no significant difference in the degree of *CDO1* methylation and the location of the tumor (duodenum, jejunum, or ileum) was found (Table 1, Fig 2).

**Table 1:** Relationship with TaqMeth V of CDO1 and clinicopathological factors at cancer tissue.

Clinicopathological factors	Compare items	n	Average of TaqMeth V	*P*-value
Age	≤60	31	66.0±53.3	0.85
	60<	22	63.2±56.6	
Gender	Male	36	72.0±51.8	0.17
	Female	17	49.7±57.5	
Histological type	Differentiated type	39	70.7±59.5	0.19
	Undifferentiated type	14	48.6±31.8	
Tumor location	Duodenum	32	65.7±63.1	0.99
	Jejunum	11	63.1±31.7	
	Ileum	10	63.9±44.8	
Tumor diameter (cm) ^※^	≤3.5	24	37.5±23.7	0.0002
	3.5<	26	92.7±62.3	
Liver metastasis	Negative	44	66.4±58.1	0.64
	Positive	9	57.1±28.7	
pT factor	T1	16	70.1±70.5	0.86
	T2	0	0	
	T3	11	58.1±48.7	
	T4	26	64.5±46.1	
pN factor	N0	30	69.3±60.4	0.72
	N1	15	55.3±37.8	
	N2	8	66.1±59.4	
pM factor	M0	37	67.4±56.4	0.60
	M1	16	58.9±49.7	
pStage	1	16	70.1±70.5	0.64
	2	10	80.1±51.5	
	3	11	60.0±34.4	
	4	16	58.9±49.7	
Lymphatic invasion	0	17	66.4±65.7	0.89
	1	36	64.1±48.8	
Venous invasion	0	17	65.5±70.3	0.95
	1	36	64.5±45.8	

^※^Three cases were unable to evaluate.

### CDO1 immunostaining of small intestine tissue

**Fig 3:**
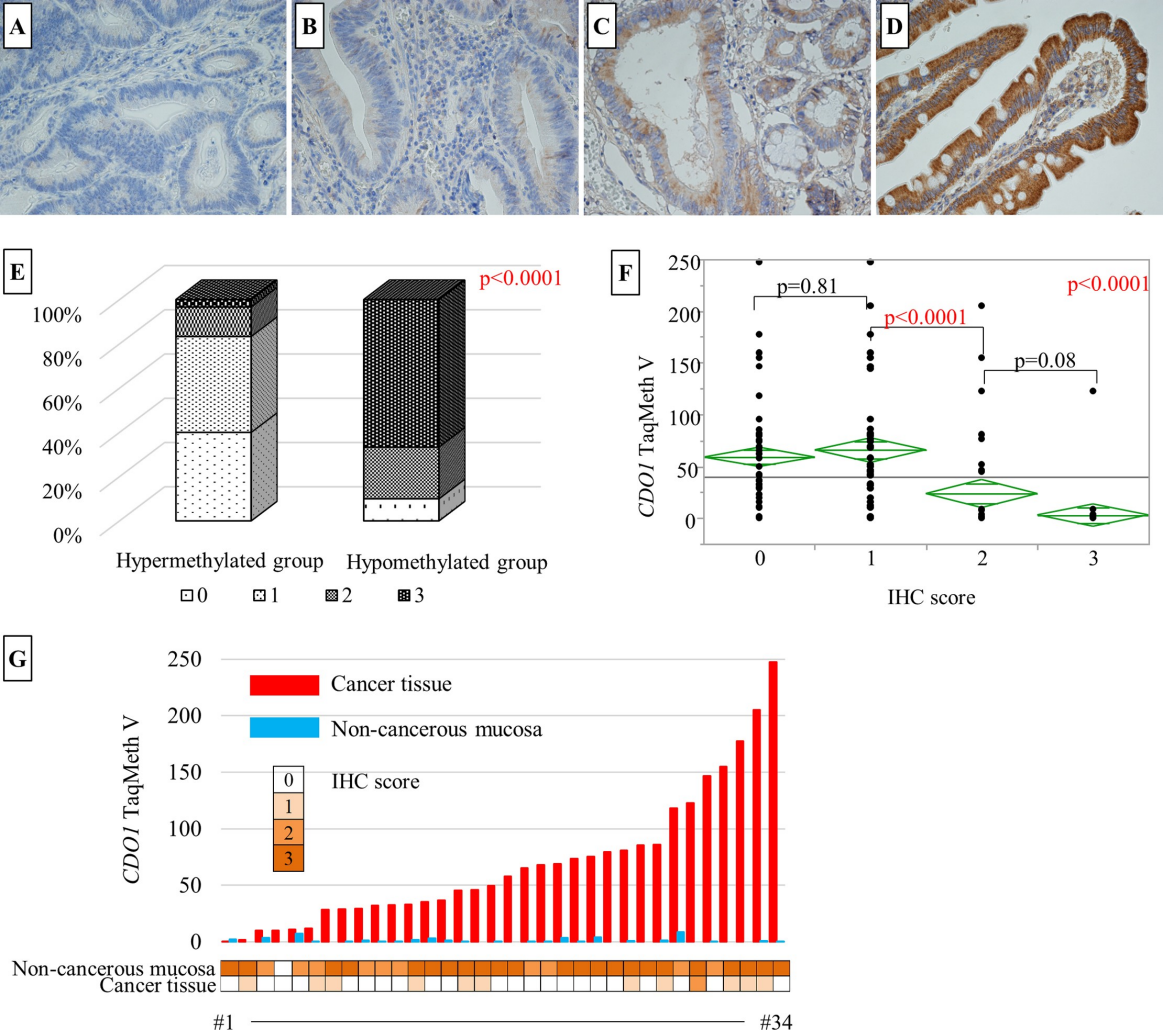
Results of CDO1 immunostaining of small bowel tissue. Immunohistochemical staining results (x400) of small bowel tissue with IHC score 0 (**A**), IHC score 1+ (**B**), IHC score 2+ (**C**), and IHC score 3+ (**D**). **E**: Tissues in the hypomethylated *CDO1* group that exhibited high CDO1 expression were observed. On the other hand, tissues in the hypermethylated *CDO1* group that exhibited low CDO1 expression were also observed. **F**: Relationship between *CDO1* TaqMeth V and IHC score. A significant difference was observed between IHC score = 1 and IHC score = 2. **G**: Relationship between TaqMath V and IHC score in 34 cases that can be evaluated in pairs.

Ten hypomethylation samples (TaqMeth V = 0.1±0.2; all samples were non-cancerous mucosa) and 10 hypermethylation samples (TaqMethV = 156.3±4.9; all samples were cancer tissues) were used for CDO1 immunostaining. From the degree of immunostaining, the evaluation was classified into four categories: no staining (IHC score = 0), dye stained lightly and whose staining remains less than 50% (IHC score = 1+), dye stained lightly and whose dyeing accounts for more than 50% (IHC score = 2+), densely stained in diffuse cells (IHC score = 3+). In the hypomethylated group, IHC score = 3+ was 67%, IHC score = 2+ was 23% and IHC score = 0 was 10%. In the hypermethylated group, IHC score = 0 was 40%, IHC score = 1+ was 43%, IHC score = 2+ was 13% and IHC score = 3+ was 3%. There was a significant difference in the degree of CDO1 immunostaining between the two groups. Especially, high immunostaining was observed in the low methylation group (Fig 3).

Since negative correlation was found between the degree of staining and TaqMeth V, immunostaining was performed also on the rest of the SBC tissues (43 samples) and the non-cancerous mucosa (24 samples). The degree of staining was compared between SBC tissues and non-cancerous mucosa. Significantly high staining was observed in non-cancerous mucosa (P <0.0001). Next, comparing the relationship between IHC score and TaqMeth V, TaqMeth V became low as the IHC score increased (p <0.0001) (Fig 3). There was a significant difference in TaqMeth V between IHC score 0–1 and 2–3 (p <0.0001). Therefore, the cutoff value of TaqMeth V, which can distinguish the staining intensity into 2 groups (0–1/2–3), was calculated using the ROC curve. As a result, the best optimized cutoff value of TaqMeth V was 9.94 (AUC = 0.88; Sensitivity 91.3%; Specificity 90.2%). This result indicated that suppressed expression of CDO1 protein is consistent with hypermethylation of *CDO1*.

Next, we compared the relationship between TaqMath V and IHC score in 34 pairs of non-cancerous mucosa and cancer tissues available in pairs. In the cancer specimen, one case had a strongly stained IHC score 2–3. In non-cancerous mucosa, one case was showing weakly stained IHC score 0–1. Most of the specimens showed staining to be inversely proportional to methylation. No special features were found for each case exhibiting atypical staining.

### Prognostic analysis of SBC

To determine if the *CDO1* TaqMeth V of SBC is a prognostic factor, Kaplan-Meier curves of OS and RFS according to the TaqMeth V were constructed and, using the log-rank plot method, appropriate cutoff values were obtained for OS and RFS. An appropriate cutoff value was not obtained in OS. However, in RFS, when the cutoff value of the TaqMeth V was 28.9, the high TaqMeth V group showed a tendency towards poor prognosis (p = 0.06, relative risk = 1.87). In addition, when the association of tumor diameter with the *CDO1* TaqMeth V was considered, the cutoff value reflecting the most dismal prognosis was 2.8 cm.

We first performed univariate analysis of prognostic factors in OS using clinicopathologic factors and found that there was a significant difference in the histological type (p = 0.005), the tumor site (p = 0.001), pT [~ submucosa (sm) vs. muscle layer (mp) ~] (p < 0.0001), pN (p = 0.02), pM (p < 0.0001), pStage (p < 0.0001), ly (p = 0.007) and v (p = 0.009). Tumor diameter (cutoff value, 2.8 cm) showed as marginally significant (p = 0.06). Since T, N and M are constituent factors of pStage, pStage was used for subsequent analysis. Analysis of the tumor diameter and the factors that showed a significant difference in univariate prognostic analysis with a multivariate Cox proportional hazard model indicated that pStage was an independent prognostic factor of OS (p = 0.04) (S3 Table).

We next performed analysis of prognostic factors in RFS, excluding distant metastasis and focusing on pStage 0 –III. Univariate analysis showed a significant difference for histological type (p = 0.02), tumor position (p = 0.01), pT (p = 0.008), pN (p = 0.003), pStage (p = 0.04) and v (p = 0.04). Ly (p = 0.05) and the *CDO1* TaqMeth V (p = 0.06) showed a marginally significant difference. Because pT and pN were included in pStage, pStage was used in subsequent analysis. No independent prognostic factors were obtained in multivariate analysis with factors showing significant or marginal differences in univariate analysis. However, the *CDO1* TaqMeth V showed marginal significance (p = 0.09) (Table 2).

**Table 2:** Univariate and multivariate prognostic analysis of clinicopathological factors for RFS in small intestinal cancer.

Clinicopathological parameters	Categories	account	Univariate analysis		Multivariate analysis		
			5year OS(%)	*p*-value	Hazard ratio	95%CI	*p*-value^※^
Age	≤ 60	21	71.7	0.91	—		
	60 <	16	71.43				
Gender	Male	13	80.5	0.15	—		
	Female	24	54.6				
Histological type	Differentiated type	30	81.8	0.02	1		0.39
	Undifferentiated type	7	25.0		2.7	0.27–27.76	
Tumor location	Duodenum	26	89.2	0.01	1		0.28
	Jejunum	3	50.0		2.9	0.10–67.18	
	Ileum	8	33.3		5	0.66–60.51	
Tumor diameter (cm)	≤2.8	14	90.0	0.39	—		
	2.8<	39	58.8				
Depth of tumor invasion (pathological)	~sm	16	100.0	0.008	—		
	mp~	21	51.5				
Lymph node metastasis (pathological)	Negative	26	83.1	0.03	—		
	Positive	11	44.4				
pStage	I	16	88.9	0.04	1		0.11
	II	10	60.0		13.5	0.25–2012.82	
	III	11	44.4		28.7	0.92–1542.52	
Lymphatic permeation	0	16	91.7	0.05	1		0.60
	1	21	57.6		1.9	0.14–26.13	
Vascular permeation	0	16	91.7	0.04	1		0.21
	1	21	57.2		6.2	0.33–150.73	
CDO1 TaqMeth V	≤28.9	10	100.0	0.06	1		0.09
	28.9<	27	57.7		9.1	0.75–267.33	

^※^Cox proportional-hazards model

### Comparison of survival and the *CDO1* TaqMeth V between SBC and CRC

**Fig 4:**
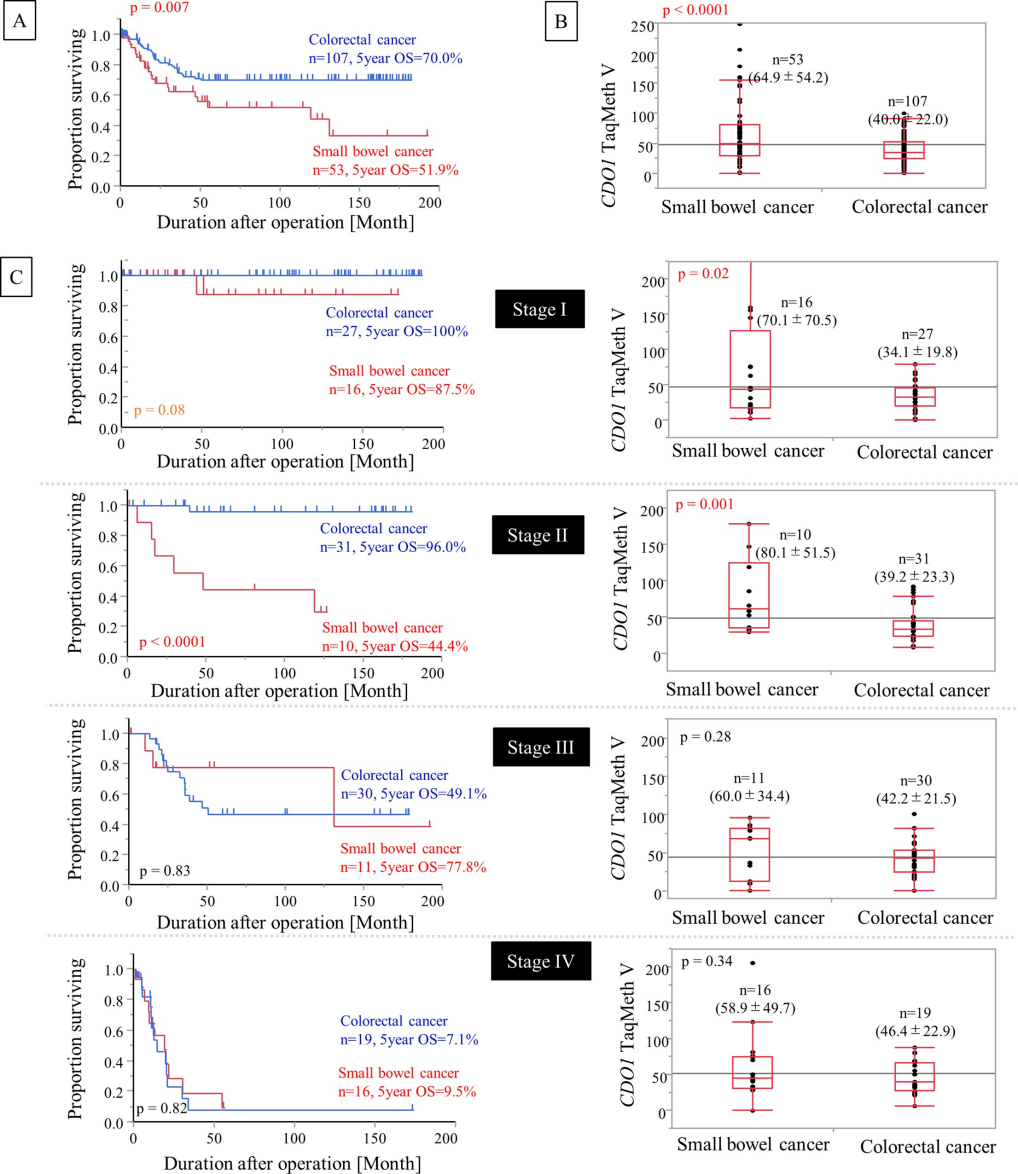
Comparison of *CDO1* methylation and prognosis between small bowel cancer and colorectal cancer. Comparison of prognosis (**A**) and *CDO1* methylation (**B**) between small bowel cancer and colorectal cancer. Compared to colorectal cancer, small bowel cancer showed significantly higher methylation and significantly poorer prognosis. (**C**) Comparison between small bowel cancer and colorectal cancer of prognosis and TaqMeth V for each Stage. In small bowel cancer, Stage I and II displayed poor prognosis and a high TaqMeth V.

The 5-year OS for total SBC was 51.9%. Comparison of OS between total SBC and total CRC showed that SBC had a significantly poorer prognosis (p = 0.007) (Fig 4A). When OS was compared corresponding to each Stage, the p value was 0.08 in pStage I, < 0.0001 in pStage II, 0.83 in pStage III, and 0.82 in pStage IV.

Next, the TaqMeth V of total SBC and total CRC was compared. The TaqMeth V of CRC was 40.0 ± 22.0, whereas as described above, the TaqMeth V of SBC was 64.9 ± 54.2. The TaqMeth V of SBC was significantly higher than that of colon cancer (p < 0.0001). When the TaqMeth V of each pStage of SBC and colorectal cancer was compared as in the prognostic analysis, p = 0.02 in pStage 0-I and the value in pStage II was significantly higher at p = 0.001. pStage III and pStage IV showed no significant difference (Fig 4B and 4C).

We randomly selected 10 cases from each stage in colorectal cancer and immunostained those specimens. There was no significant difference in the degree of staining between SBC and CRC (p = 0.09). In addition, the degree of staining for each stage in SBC and CRC was compared. The p-value at each stage was p = 0.16 for Stage I, p = 0.33 for Stage II, p = 0.32 for Stage III, and p = 0.38 for Stage IV. There was no significant difference between the organizations in each stage comparison.

### Comparison of the TaqMeth V among small bowel tumors

**Fig 5:**
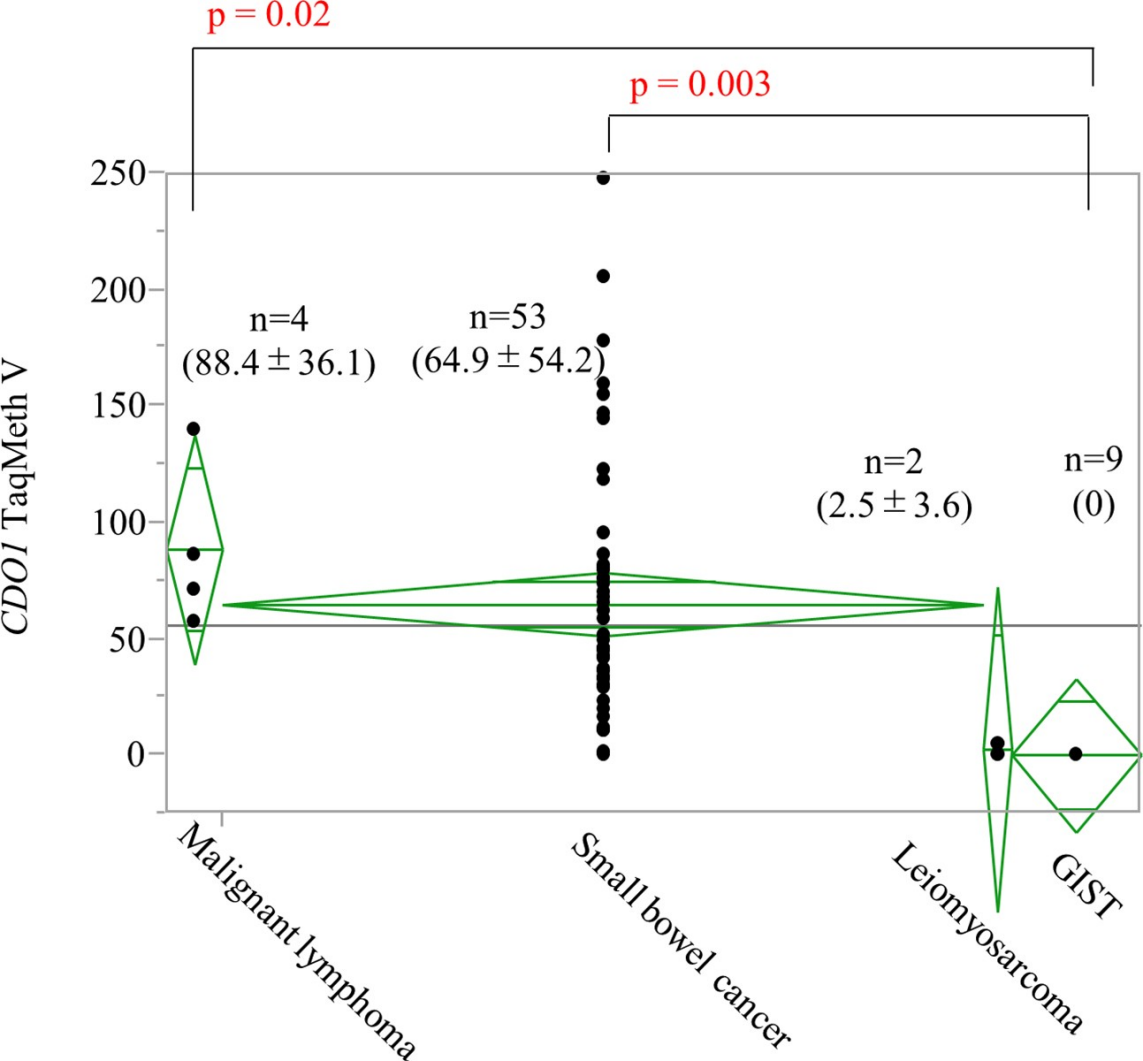
Comparison of the TaqMeth V among small bowel tumors. The TaqMeth V for the indicated small bowel tumors is shown. Small bowel cancer and malignant lymphoma showed high CDO1 methylation. On the other hand, leiomyosarcoma and GIST were hypomethylated.

The TaqMeth V of the different small bowel tumors was 88.4 ± 36.1 for malignant lymphoma, 2.5 ± 3.6 for leiomyosarcoma, and 0 for GIST. In the comparison of TaqMeth V among tumors including SBC, malignant lymphoma showed high *CDO1* methylation along with SBC, whereas *CDO1* methylation in GIST was significantly lower than that of these two tumors (p = 0.002) (Fig 5).

CDO1 immunostaining was performed on small bowel tumors. Three specimens of malignant lymphoma, two of leiomyosarcoma, and nine of GIST were available for immunostaining. There was no significant difference in the degree of staining between tissues (p = 0.18).

## Discussion

SBC is a disease that is significantly rarer than other gastrointestinal cancers [1]. However, with the recent improvements in diagnostic techniques, the frequency of clinical treatment of SBC has increased. In clinical practice, treatment is usually performed according to the treatment of CRC, although sometimes it is according to the treatment of gastric cancer. While small intestine cancer has been indicated to be similar to CRC, studies on its carcinogenesis mechanism are still insufficient. This study is the first report regarding the relationship between methylation abnormality of the *CDO1* gene and SBC.

We first compared methylation abnormality of the *CDO1* between SBC tissue and small intestine non-cancerous mucosa by using the TaqMeth V. Significantly high *CDO1* methylation abnormality was observed in the cancer tissue, and a TaqMeth V = 4.09 was obtained as the cutoff value. We can infer from this result that methylation abnormality of the *CDO1* is strongly involved in the carcinogenesis of SBC. There have been reports of cancer-specific *CDO1* methylation abnormalities in the gastrointestinal tract in esophageal cancer, stomach cancer and CRC [15, 17–20]. The results of this study newly showed that cancer-specific *CDO1* methylation abnormality is found even in SBC.

We evaluated the expression of the CDO1 protein using immunostaining to determine how methylation of the *CDO1* is involved in expression of the CDO1 protein. We extracted ten low TaqMethV specimens and 10 high TaqMethV specimens and compared CDO1 expression with immunostaining. Certainly, immunostaining was strongly observed in the hypomethylated group, and weakly in the hypermethylated group. From the immunostaining results, it was confirmed that the expression of CDO1 protein is under the control of methylation. This result was similar to the results we have obtained in the esophageal squamous cell carcinoma, gall bladder cancer and CRC so far [17, 26, 27]. But, even among the high contrasting groups, 10% of hypomethylated group shown no immunostaining and 16% of hypermethylated demonstrated immunostaining. It can be said that even two groups with the greatest difference in TaqMeth V showed contradiction by immunostaining. This contradiction can be interpreted as follows. One reason for this lack of correlation in several SBC is because clinical specimens were used in the present study. Thus, it was speculated that CDO1 expression was maintained by the transcriptional activity of the remaining unmethylated allele, even though the other allele was undergoing dense methylation in the cancer tissue. This finding is remarkable in nonepithelial malignant tumors of the small intestine. From this view, in order to evaluate the methylation status of DNA and the contribution to gene expression from both alleles, we confirmed the IHC score of 34 cases that can be evaluated in pairs. However, we could not find a clear relationship in the examination of pairs of non-cancerous mucosa and cancer tissues. From this result, we think that the influence of the possibility of PCR assay with its intrinsic technical issues is considered as another reason. We used actual clinical specimens, therefore, these results were considered to be the theoretical difficulty of perfect match.

We next analyzed the clinicopathological features that might be associated with methylation abnormality of the *CDO1*. There was little association between *CDO1* methylation and the degree of progression of SBC or the site where the tumor existed. When a tumor diameter of 3.5 cm was taken as the cutoff value, high methylation was shown to depend on increased tumor size. Focusing on the stage, it was found that pStage I already exhibited advanced *CDO1* methylation, which reflects the fact that the disease of SBC is generally considered to have a poor prognosis. This result was consistent with the characteristic of methylation abnormality of the *CDO1* in Barrett's esophageal cancer that is clinically malignant [20]. Moreover, it has been reported that *CDO1* is involved in tumor cell growth, cell migration, invasion, and the ability of colony formation [28]. In this study, we can provide clinical evidence to support the possibility that methylation of *CDO1* is involved in tumor cell proliferation. Since there have also been some reports that *CDO1* methylation is related to prognosis [17, 21], the involvement of *CDO1* methylation in the prognosis of SBC was also analyzed. When calculating the cutoff value of TaqMeth V using the log-rank plot method, an appropriate cutoff value (TaqMeth V, 28.9) was obtained in RFS. In multivariate analysis, although the TaqMeth V was not shown to be an independent prognostic factor, it did show marginal significance in RFS. We showed the possibility that methylation abnormality of *CDO1* could contribute to prognosis as a recurrence risk factor. In OS, pStage became an independent prognostic factor, which is consistent with published results [8, 29].

DNA methylation is a stable modification that has attracted attention as a disease marker due to its stability [9, 30]. Unlike the case for gastric cancer and CRC, there is no screening test for SBC in clinical practice. High methylation abnormality of the *CDO1* was from the early stage of SBC. In addition, the *CDO1* TaqMeth V was possibly a risk factor of recurrence. These findings indicated that methylation abnormality of *CDO1* has high clinical significance as a useful biomarker of SBC. We would have investigated *CDO1* methylation status in precancerous lesions of SBC in order to demonstrate the utility as a biomarker of methylation abnormality of *CDO1*. The results showed that methylation increased with advancing atypia (non-cancerous mucosa <adenoma <SBC tissue). In our previous reports [27], we also report the association between *CDO1* methylation abnormality and atypia of CRC. The carcinogenic form of SBC may be similar to CRC. As a result, utilization of *CDO1* methylation abnormality can be expected as a biomarker for detection from adenoma to SBC.

In past reports on SBC, most studies of risk factors and genetics were performed with reference to CRC. Crohn's disease, celiac disease, Lynch syndrome, familial adenomatous polyposis, and Peutz-Jeghers syndrome have been indicated as risk factors for SBC [1, 31–35]. In SBC, as in CRC, the mechanism of carcinogenesis of adenoma-carcinoma sequence has been pointed out [36]. In this study, compared to colorectal cancer, the prognosis of SBC was significantly poorer; in particular Stages of SBC up to pStage II had poor prognosis. This result can be said to indicate the clinical malignancy of SBC. Furthermore, in comparison of the *CDO1* TaqMeth V between the two cancers, TaqMeth V was significantly higher in SBC up to Stage II compared with CRC. Based on these results it can be said that clinical malignancy is indicated by methylation abnormality of the *CDO1*.

Malignant lymphoma, GIST, and leiomyoma cannot be disregarded when considering small bowel tumors and therefore, methylation abnormality of *CDO1* was also investigated in these tumors. Although high *CDO1* methylation was observed in malignant lymphoma as well as in adenocarcinoma, *CDO1* methylation was extremely low in leiomyosarcoma and in GIST that is characterized by spindle shaped cells.

Factors such as microsatellite instability, p53, APC, and K-ras have been mainly investigated in the study of carcinogenesis of SBC [1,6,37–40]. Although gene methylation abnormality in SBC has also been studied, it has not yet been clinically applied. Treatment of SBC is often based on the treatment of colorectal cancer. However, its outcome is poor. As described above, there is also lack of evidence regarding what therapy to use for SBC. Therefore, early detection is indispensable for obtaining a better prognosis. In this study, we showed cancer-specific methylation of *CDO1* in SBC, its association with clinical malignancy, and its potential to function as a recurrence prediction marker. We plan to report biomarker research using methylation abnormality of the *CDO1* to accumulate evidence that might lead towards better diagnosis of SBC.

## Supporting information

S1 Table Clinicopathological characters of patients. (DOCX)

S2 Table PCR condition and primer and fluorescent probe sequences for Q-MSP. (DOCX)

S3 Table Univariate and multivariate prognostic analysis of clinicopathological factors for OS in small intestinal cancer. (DOCX)
