# Supplementary material for: Clinical significance of cancer specific methylation of the CDO1 gene in small bowel cancer
Source: PLoS One. 2019 Jan 24;14(1):e0211108. doi: 10.1371/journal.pone.0211108 (PMC6345476; doi:10.1371/journal.pone.0211108)
Supplement: Supplementary file 1 [file pone.0211108.s001.docx]

S1 Table. Clinicopathological characters of patients.

Clinicopathological parameters	Categories	Account
Age	Mean ± S.D.	60.3±12.1
Gender	Male	36
	Female	17
Histological type	Differentiated	39
	Undifferentiated	14
Tumor location	Duodenum	32
	Jejunum	11
	Ileum	10
Tumor diameter (cm) ※	Mean ± S.D.	4.8±2.3
Liver metastasis	Negative	44
	Positive	9
pT factor (7th UICC)	T1	16
	T2	0
	T3	11
	T4	26
pN factor (7th UICC)	N0	30
	N1	15
	N2	8
pM factor (7th UICC)	M0	37
	M1	16
pStage (7th UICC)	1	16
	2	10
	3	11
	4	16
Lymphatic invasion (ly) (7th UICC)	0	17
	1	36
Venous invasion (v) (7th UICC)	0	17
	1	36

※Three cases were unable to evaluate.
