# Supplementary material for: Clinical significance of cancer specific methylation of the CDO1 gene in small bowel cancer
Source: PLoS One. 2019 Jan 24;14(1):e0211108. doi: 10.1371/journal.pone.0211108 (PMC6345476; doi:10.1371/journal.pone.0211108)
Supplement: Supplementary file 2 [file pone.0211108.s002.docx]

S2 Table. PCR condition and primer and fluorescent probe sequences for Q-MSP.

Target gene	Forward primer	Fluorescent probe or internal primar	Reverse primer
**CDO1**	CCACAACGACGAAAATAAAACG	FAM-TTAACGGCGCGTTTTAGTCGTTCG-TAMRA	TCGGCGTTTTAGGGATCGCG
**β-actin**	TGGTGATGGAGGAGGTTTAGTAAGT	FAM-ACCACCACCCAACACACAATAACAAACACA-TAMRA	AACCAATAAAACCTACTCCTCCCTTAA
