# Supplementary material for: Clinical significance of cancer specific methylation of the CDO1 gene in small bowel cancer
Source: PLoS One. 2019 Jan 24;14(1):e0211108. doi: 10.1371/journal.pone.0211108 (PMC6345476; doi:10.1371/journal.pone.0211108)
Supplement: Supplementary file 3 [file pone.0211108.s003.docx]

S3 Table. Univariate and multivariate prognostic analysis of clinicopathological factors for OS in small intestinal cancer.

Clinicopathological parameters	Categories	account	Univariate analysis		Multivariate analysis	
			5year OS(%)	*p*-value	Hazard ratio	95%CI
Age	≤ 60	31	40.1	0.10	---	
	60 <	22	69.1			
Gender	Male	36	53.1	0.89	---	
	Female	17	48.1			
Histological type	Differentiated type	39	66.3	0.005	1	
	Undifferentiated type	14	11.9		3.54	0.98 - 14.89
Tumor location	Duodenum	32	68.5	0.001	1	
	Jejunum	11	13.5		5.74	1.23 - 33.60
	Ileum	10	41.1		1.89	0.45 - 7.71
Tumor diameter (cm)	≤2.8	14	87.5	0.06	1	
	2.8<	39	41.2		1.47	0.31 - 10.68
Liver metastasis	Negative	44	63.5	0.02	---	
	Positive	9	12.7			
Depth of tumor invasion (pathological)	~sm	16	87.5	0.002	---	
	mp~	37	35.9			
Lymph node metastasis (pathological)	Negative	30	66.7	0.02	---	
	Positive	23	30.6			
Distant metastasis	Negative	37	70.1	<0.0001	---	
	Positive	16	9.5			
pStage	pStage I	16	87.5	<0.0001	1	
	pStage II	10	44.4		48.58	0.63 - 4167.06
	pStage III	11	77.8		12.34	0.11 - 1046.16
	pStage IV	16	9.5		58.29	0.54 - 6319.02
Lymphatic permeation	0	17	81.7	0.007	1	
	1	36	38.3		2	0.13 - 13.20
Vascular permeation	0	17	80.0	0.009	1	
